# Population-based incidence and risk factors for cholestasis in hemolytic disease of the fetus and newborn

**DOI:** 10.1038/s41372-022-01345-1

**Published:** 2022-02-22

**Authors:** Jonas Teng, Linnéa Wickman, Marie Reilly, Antal Nemeth, Björn Fischler, Kajsa Bohlin, Eleonor Tiblad

**Affiliations:** 1 Division of Pediatrics, Department of Clinical Science, Intervention and Technology (CLINTEC), Karolinska Institutet, Stockholm, Sweden; 2 Department of Pediatrics, Södertälje Hospital, Stockholm, Sweden; 3 Center for Fetal Medicine, Pregnancy Care and Delivery, Women´s Health, Karolinska University Hospital, Stockholm, Sweden; 4 Department of Medical Epidemiology and Biostatistics, Karolinska Institutet, Stockholm, Sweden; 5 Department of Pediatrics, Karolinska University Hospital, Stockholm, Sweden; 6 Department of Neonatology, Karolinska University Hospital, Stockholm, Sweden; 7 Clinical Epidemiology Division, Department of Medicine Solna, Karolinska Institutet, Stockholm, Sweden

**Keywords:** Epidemiology, Paediatrics, Immunological disorders, Anaemia, Diagnostic markers

## Abstract

**Objective:**

To estimate the incidence of cholestasis in neonates with hemolytic disease of the fetus and newborn (HDFN) and investigate risk factors and long-term liver disease.

**Study design:**

A population-based cohort study of all infants born with HDFN within the Stockholm region between 2006 and 2015. The study period was the first 90 days of life, and presence of any chronic liver disease was evaluated at two years of age.

**Results:**

Cholestasis occurred in 7% (11/149). Median age at detection was 1.1 days. Intrauterine blood transfusions and maternal alloimmunization with multiple red blood cell antibodies including D-, c- or K-antibodies were independent risk factors for cholestasis. No infant had chronic liver disease at two years of age.

**Conclusions:**

Infants with severe HDFN have increased risk for cholestasis, particularly those requiring multiple intrauterine transfusions. Early and repeated screening for conjugated hyperbilirubinemia in the first week of life is needed to ensure adequate management.

## Introduction

Hemolytic disease of the fetus and newborn (HDFN) is caused by maternal IgG-antibodies against antigens on the fetal erythrocytes. Severe disease is most commonly due to antibodies directed against the Rhesus-D-antigen [1]. Other antibodies known to cause significant hemolysis and fetal anemia are anti-c and anti-K [2]. Since the introduction of prophylaxis for RhD-immunization the number of severe HDFN cases and subsequent intrauterine deaths have decreased considerably. This has resulted in a relative increase in other red cell immunizations, but RhD disease remains the dominant cause for severe HDFN [3]. Fetomaternal incompatibility and constitutional antibodies within the ABO-system are not included in routine prenatal screening since fetal anemia in these pregnancies is extremely rare, but nowadays these account for the major part of neonatal jaundice cases due to erythrocyte alloimmunization. A combination of more than one antibody can complicate the pregnancy and there are reports of an association with more severe HDFN [4–7]. The treatment of fetal anemia consists of intrauterine blood transfusions (IUT) and scheduled delivery to enable postnatal treatment.

In the newborn, HDFN may lead to pronounced unconjugated hyperbilirubinemia, with a risk for development of bilirubin-induced encephalopathy [8]. Almost a century ago, infants with HDFN who also developed cholestasis were described [9]. In clinical practice cholestasis is defined by conjugated hyperbilirubinemia [10–13]. The cut-off values of conjugated bilirubin (CB) used to define cholestasis vary. Most frequently, a cut-off of CB exceeding 1 mg/dL (≈17 μmol/L) or 2 mg/dL (≈ ≥34 μmol/L) is used, and sometimes definitions also require the CB to exceed 20% of the total bilirubin (TB) level [14–16].

There are different hypotheses regarding the pathogenesis of cholestasis in infants with HDFN, such as viscous bile from intense hemolysis, hepatic iron overload from IUTs, anemia and hypoxia in the liver or hepatic injury from extra-medullary erythropoiesis [12, 17, 18]. There are several case reports of cholestasis in neonates with HDFN, but only a few studies have investigated incidence, risk factors and outcomes [18, 19].

The aim of this study was to estimate the incidence of neonatal cholestasis in a population-based cohort of infants with HDFN, to identify important risk factors and prevalence of long-term chronic liver disease.

## Subjects and methods

### Study cohort

The study cohort consists of all live born children with hyperbilirubinemia caused by HDFN identified in the population of alloimmunized pregnancies in the Stockholm region during a ten-year period from January 2006 to December 2015.

In the Stockholm region, all pregnant women are offered screening for irregular red cell antibodies in late first trimester. RhD negative women are screened a second time, in early third trimester. All positive screening tests and titration results for red cell antibodies during pregnancy are registered at the regional transfusion medicine center at Karolinska University Hospital. All pregnancies with antibodies are then routinely registered into the GravImm register (www.gravimm.se). In addition to data from the transfusion medicine center, intrauterine blood transfusions, pregnancy outcomes and neonatal outcomes are collected in this register, together with additional details from obstetric and pediatric medical records. All women with red cell alloantibodies were managed at Karolinska University Hospital. From 2008 and onwards all data were recorded prospectively in the GravImm register, and prior records were entered retrospectively.

Inclusion criteria were live-born infants to mothers in the GravImm register, where the infant had received a diagnosis of HDFN (ICD10 codes P55.0, P55.8 or P55.9). Infants who had no conjugated bilirubin levels analyzed postnatally were excluded. There were no post hoc exclusion criteria. Since the study uses observational data that was collected retrospectively from the GravImm register and medical charts, this investigation reflects clinical practice and not a specific protocol for the management of the infants with HDFN.

### Data sources

Maternal and pregnancy details were obtained from the GravImm register and obstetric charts. From all the types of maternal antibodies that were recorded, we created variables to denote if a specific antibody was present alone or in combination with at least one other antibody (multiple antibodies).

Information about the neonates, their postnatal care and treatments, was obtained from medical records. The results for every available serum analysis of bilirubin, liver enzymes and bile acids levels during the first 90 days of life were extracted from laboratory records together with the time and date of the assay. Medical charts of all infants were reviewed up to two years of age to identify children who had any diagnosis of chronic liver disease.

### Intrauterine blood transfusions

Cases with severe fetal anemia, defined as a maximum peak systolic velocity in the middle cerebral artery >1.5 multiples of the median [20], were treated with IUT to the fetus. All IUTs were performed at the Center for Fetal Medicine at Karolinska University Hospital as previously described and no important changes in the protocol were made during the study period [21].

### Definitions of outcomes

Cholestasis was defined as serum CB exceeding 34 μmol/L (≈2 mg/dL) and exceeding 20% of serum TB. Three other common definitions of cholestasis were also explored: CB ≥ 17 μmol/L; CB ≥ 17 μmol/L and ≥20% of TB; CB ≥ 34 μmol/L.

The postnatal age when first identified as fulfilling the criteria for these diagnoses was examined as a “time-to-event” outcome. Any cholestasis diagnosis was analyzed as a dichotomous outcome. Liver disease of clinical significance by two years of age was defined as enrollment with a diagnosis of liver disease at the regional referral center for pediatric hepatology, at Karolinska University Hospital, or at any other pediatric clinic in the Stockholm region.

### Statistical analysis

The time to detection of cholestasis using the four different definitions was investigated using Kaplan–Meier analysis and the cumulative incidence presented graphically. Infants with cholestasis were compared to non-cholestatic infants for maternal, prenatal and neonatal factors. Fisher exact tests were used to compare proportions, Mann–Whitney *U* tests to compare medians, two-tailed Student *t* tests for means and Spearman correlation coefficients for correlations. All variables with a significant crude association with cholestasis were further investigated for their independent association by including them in a multivariable logistic regression model together with the antibody status with strongest crude association.

SPSS for Mac version 26 (IBM, New York, USA) was used. Statistical significance level was set at 5%.

## Results

### Cumulative incidence of cholestasis

From the GravImm register search, 211 infants were assessed for eligibility. Out of these, 41 were not eligible due to being born or treated in other regions (*n* = 9), initial positive antibody titer that later in pregnancy was negative (*n* = 4), HDFN not requiring treatment (*n* = 13) or father or infant negative for antigen (*n* = 15), see study flow chart in Supplementary Fig. 1. Eligible for the study cohort consisted of 170 infants with hyperbilirubinemia requiring treatment caused by HDFN. Within the Stockholm region during the study period, there were 28 019 live births on average each year. Relative to the total of 280 187 live births in the region during the study period [22], the incidence of HDFN requiring treatment was 0.6 per 1000 live-born infants.

Of the 170 infants, 21 had never been tested for CB levels and the remaining 149 were included in the analysis, see study flow chart in Supplementary Fig. 1. RhD was the most common immunization, affecting 63% of the pregnancies. The distribution of primary antibodies is presented in Table 1. None of the infants were affected by hydrops at birth.

**Table 1:** Distribution of primary antibodies.

Primary antibody^a^	Number	Percent
D	94	63.1
c	27	18.1
E	10	6.7
Fy(a)	4	2.7
U	4	2.7
K	3	2.0
C	2	1.3
C(w)	1	0.7
e	1	0.7
Jk(b)	1	0.7
M	1	0.7
S	1	0.7
Total	149	100.0

^a^When multiple antibodies were present, the one with the highest titer was defined as primary.

Eleven out of 149 (7%) of the neonates developed cholestasis. The age at which the main cholestasis criteria were fulfilled ranged from 0 to 45 days, with a median of 1.1 days. The time to detection using the different definitions for cholestasis is displayed in Supplementary Fig. 2, showing that cholestasis was detected in the first week of life in most cases, regardless of which definition was used. The cumulative incidence was considerably higher when using definitions not including the criterion for CB to exceed 20% of TB.

### Comparison of infants with and without cholestasis

For the infants classified as cholestatic (CB ≥ 34 μmol/L and ≥20% of TB) and non-cholestatic, Table 2 compares baseline characteristics and maternal antibody status, pre-and postnatal treatments and bilirubin levels. No significant differences were observed for the number of previous pregnancies, parity, live births or intrauterine fetal deaths (data not shown). There was a significant difference in gestational age and birth weight between the two groups, both being lower in the cholestasis group. IUT had been performed in 9 out of 11 (82%) pregnancies where the infant developed cholestasis, as compared to 24 out of 138 (17%) pregnancies in the non-cholestatic group (*p* < 0.0001). Among those treated, the median number of IUTs was 3 in both groups, with a range of 1–9 transfusions in the cholestatic group and 1–6 in the non-cholestatic group. There was no significant difference in the median gestational age at the first IUT: 28 weeks and 2 days (range 19 + 0–33 + 0) versus 30 weeks and 2 days (range 20 + 6–35 + 5) (*p* = 0.54, Mann–Whitney *U*-test). Apgar score at 5 min of age ranged from 6 to 10 among non-cholestatic infants and 5 to 10 in cholestatic (*p* = 0.22), and no case of cholestasis was considered to be due to perinatal asphyxia. Culture-verified sepsis occurred in two of the non-cholestatic infants. Necrotizing enterocolitis affected one infant in the cholestasis group after the resolution of cholestasis, which did not reappear. All infants, both cholestatic and non-cholestatic, received phototherapy in the neonatal period, but none developed bronze baby syndrome. None of the infants in either group died within the first 3 months of life. The maternal red cell antibodies most strongly associated with cholestasis were anti- D-, c- or K-antibodies in combination with red cell antibodies of any type, i.e., multiple antibodies where at least one of anti-D, -c or -K were present.

**Table 2:** Characteristics of cholestatic and non-cholestatic infants and their mothers.

	Cholestatic (*n* = 11)^a^	Non-cholestatic (*n* = 138)^a^	*p*^b^
Baseline characteristics			
Age of mother at birth, years, mean (SD)	32.2 (5.3)	33.4 (4.5)	0.39
Vaginal delivery	4/11 (37%)	92/138 (67%)	0.05
Gender, female	5/11 (46%)	58/138 (42%)	0.53
Gestational age at birth, weeks + days, median (range)	36 + 0 (29 + 6–37 + 2)	37 + 2 (28 + 6–40 + 6)	0.002^c^
Birth weight, g, mean (SD)	2648 (699)	3124 (649)	0.02^c^
Any incompatibility in ABO-system	1/9 (11%)	21/135 (16%)	1.0
Maternal alloantibodies			
D immunization, primary	8/11 (73%)	86/138 (62%)	0.75
D immunization with multiple antibodies	6/11 (55%)	18/138 (13%)	0.002^c^
D, c or K immunization, primary	10/11 (91%)	114/138 (83%)	0.69
D, c or K immunization, with multiple antibodies	8/11 (73%)	24/138 (17%)	<0.001^c^
Pre- and postnatal treatments			
Any intrauterine transfusion	9/11 (82%)	24/138 (17%)	<0.001^c^
Exchange transfusions	7/11 (64%)	27/138 (20%)	0.003^c^
IVIG treatment	4/11 (36%)	16/138 (12%)	0.04^c^
Phototherapy, hours of treatment, median (range)	81 (13–125)	68 (6–610)	0.68
Postnatal erythrocyte transfusions	9/11 (82%)	30/138 (22%)	<0.001^c^
Any parenteral nutrition	2/11 (18%)	8/138 (6%)	0.16
Ursodeoxycholic acid treatment	6/11 (55%)	0/138 (0%)	<0.001^c^
Jaundice and cholestasis			
Peak TB, μmol/L, mean (SD)	333.2 (99.0)	287.0 (63.4)	0.16
Age at peak TB, days, mean (SD)	3.13 (1.7)	4.43 (2.9)	0.14
Peak CB, μmol/L, mean (SD) *– grouping variable*	155.7 (121.2)	20.2 (10.9)	0.004^c^
Age at peak CB, mean (SD)	9.1 (13.2)	5.8 (9.1)	0.26

*SD* standard deviation, *IVIG* intravenous immunoglobulin, *TB* total bilirubin, *CB* conjugated bilirubin.

^a^n/N (%) unless otherwise specified.

^b^Independent samples *t*-test (2-tailed) for means, Fisher exact test for proportions, Mann–Whitney *U*-test when medians reported.

^c^Significance at 0.05-level.

Detailed individual descriptive data regarding the cholestatic cases are presented in Table 3. Serum ferritin level was measured in all but one infant, and all had elevated levels, including the two infants who had not received any IUT. The postnatal peak ferritin levels were strongly correlated to the number of IUTs given prenatally, with a Spearman correlation coefficient 0.79 (*p* = 0.007). Six out of eleven infants had peak CB levels exceeding 100 μmol/L. These infants also received ursodeoxycholic acid and vitamin K treatment. The peak ferritin and peak CB levels correlated to a high degree, with a Spearman correlation coefficient of 0.78 (*p* = 0.008). In four cases, serum bile acids were analyzed and all found to be elevated above the laboratory reference level (range 18–126 μmol/L, reference <10), but tests were performed at different times later in the cholestatic course, after the peak CB level had passed. Ultrasound of the liver and bile ducts was performed in 5/11 of the cholestatic infants, all with normal findings. In one of these cases, the ultrasound showed an enlarged liver at 2 days of age, but this had normalized by 18 days.

**Table 3:** Detailed characteristics of cholestatic cases.

Case	Antibody, primary (secondary)	Gender	Gestational age, week + day	Birth weight, g	Apgar score at 5 min age	Intrauterine RBC transfusions	Exchange transfusions^a^	Postnatal RBC transfusions	Peak conjugated bilirubin (μmol/L)	Age, days, when CB < 34 μmol/L	Peak Ferritin (μg/L)^b^	Peak ALT/AST (µkat/L)^c^	Peak GT (µkat/L)^c^	Peak INR^d^	UDCA and Vitamin K
1	K (D)	M	34 + 3	2690	5	5	0	0	90	48	2000	0.99/1.47	1.2	1.1	N
2	D (C)	F	36 + 6	2762	10	9	0	1	248	63	11893	5.7/8.3	–	1.3	Y
3	D	M	36 + 2	3380	10	2	2	0	315	33	4010	2.1/4.1	0.86	1.6	Y
4	D	M	35 + 5	3000	9	2	3	1	126	9	624	0.38/0.45	1.5	1.5	Y
5	E (c)	F	36 + 1	2595	10	0	1	1	138	11	558	0.59/0.63	3.3	1.2	Y
6	D	M	29 + 6	1961	6	2	4	2	51	6	808	1.01/2.19	1.5	1.5	N
7	D (C, S)	F	36 + 4	3025	10	3	1	1	35	60	614	1.95/1.35	1.7	–	N
8	D (C, Fy(a))	F	36 + 0	2520	10	7	1	6	412	71	9240	7.94/8.52	1.3	1.4	Y
9	c (E)	M	37 + 2	3880	10	0	0	2	48	15	470	0.20/0.66	2.4	1.3	N
10	D (C)	M	34 + 0	1430	9	3	2	4	157	123	2866	3.74/2.98	7.8	1.3	Y
11	D (C)	F	35 + 2	1890	10	1	0	1	88	44	–	0.27/0.90	9.2	–	N

*RBC* red blood cell, *IVIG* intravenous immune globulin, *ALT* alanine aminotransferase, *AST* aspartate aminotransferase, *GT* gamma-glutamyl transferase, *INR* international normalized ratio; UDCA, ursodeoxycholic acid.

^a^All infants received phototherapy and none developed bronzing.

^b^Reference <350 μg/L.

^c^Reference <0.5 µkat/L (1 µkat/L = 60 IU/L).

^d^Reference <1.2.

A subgroup analysis was performed comparing the seven infants with early detection of cholestasis, during the first 48 h of life, with those four infants detected later. The groups did not differ significantly except for variables regarding the severity of cholestasis. The median peak CB level was 157 μmol/L (range 48–412 μmol/L) in the early detection group, versus 69.5 μmol/L (range 35–90 μmol/L) in the late detection group (*p* = 0.038). In the early detection group, six out of seven received UDCA and vitamin K treatment, versus none of the four in the late group (*p* = 0.015).

### Risk factors for cholestasis

The results from the multivariable logistic regression analysis are presented in Table 4. The maternal red cell antibody status most strongly associated with cholestasis (immunization with D-, c- or K-antibodies in combination with red cell antibodies of any type) and other potential risk factors available at or before birth, were first analyzed separately using binary logistic regression, and variables with a significant crude association with cholestasis are presented in the first column in Table 4 (detailed results are presented in Supplementary Table S1). Variables with a significant crude association were entered in a multivariable logistic regression model to assess their independent associations. Intrauterine transfusions and maternal immunization with D-, c- or K-antibodies in combination with red cell antibodies of any type, remained as independent risk factors, but there was no evidence of an independent association with GA and birth weight (Table 4).

**Table 4:** Risk factors for cholestasis.

	Crude OR (95% CI)	*p*	Adjusted OR (95% CI)	*p*
Gestational age^a^	0.75 (0.60–0.94)	0.014*	0.75 (0.40–1.39)	0.35
IUT treatment^b^	21.4 (4.3–105.3)	<0.001*	17.4 (3.0–100.9)	0.002*
Birth weight^c^	0.999 (0.998–1.000)	0.025*	1.001 (0.999–1.003)	0.58
D, c or K immunization, with multiple antibodies^d^	12.7 (3.1–51.2)	<0.001*	10.7 (2.2–53.3)	0.004*

Binary logistic regression.

*OR* odds ratio, *IUT* intrauterine transfusion.

^a^Per additional week of gestational age.

^b^Reference: no IUT treatment.

^c^Per additional gram.

^d^Reference: all other antibody statuses.

*Statistical significance at 0.05-level.

### Long-term follow-up

Of the 149 children in the study, 12 were lost to follow-up due to moving abroad or to other regions (2 in the cholestatic and 10 in the non-cholestatic group) leaving 137 (92%) available for follow-up.

By two years of age, none of the cholestatic (*n* = 9) or non-cholestatic infants (*n* = 128) had developed chronic liver disease. However, one cholestatic infant, who had received 3 IUTs due to maternal D- and C-antibodies, underwent laparotomy at 4 months of age. Preoperative percutaneous liver biopsy showed cholestasis and bile duct proliferation. These findings, in combination with lack of excretion on hepatobiliary scintigraphy were considered to be compatible with biliary atresia. However, biliary ducts were normal on perioperative cholangiography. The cholestasis had resolved at five months of age, but elevated transaminases remained until 1.5 years of age. A follow-up liver biopsy showed normal findings.

## Discussion

Our main finding in this population-based cohort study was that cholestasis is common in infants with HDFN and that it is present at birth or developed shortly afterward. The risk for cholestasis was associated with prenatal IUT and maternal D-, c- or K-antibodies in combination with other red cell antibodies, but was not independently associated with preterm birth. Our interpretation of the findings is that a more severe hemolytic disease increases the risk of cholestasis. We also found that although HDFN-associated cholestasis may be severe, it was most commonly transient and not associated with chronic liver disease at two years of age. Even though we did not perform any examination of the study subjects during this time, any child with clinically apparent liver diseases would have been referred to the regional referral center for pediatric hepatology at the Karolinska University hospital, and thereby identified in our study.

The cumulative incidence of cholestasis was 7% in our cohort. In the only larger study of cholestasis in infants with HDFN, a group from the Netherlands found an incidence of 13% in term or near term infants with HDFN, and reported D-immunization and IUT as independent risk factors [18]. Our results are in line with this study, since by using the same definition of cholestasis (CB ≥ 17 μmol/L and ≥20 % of total), the cumulative incidence was 10% in our cohort. A study from Turkey reported a 60% incidence of cholestasis among infants with fetal hydrops, i.e., selecting the most severe cases of HDFN [19]. While these infants are not fully comparable to our cohort, their findings support our reasoning that a more severe HDFN increases the risk for cholestasis.

Different cut-off levels for conjugated bilirubin have been used when defining neonatal cholestasis. Regarding the absolute level of CB, setting it as low as 17 μmol/L (1 mg/dL) is more inclusive, minimizing the risk of missing a potential case. However, this may also include infants who would not be perceived as cholestatic by most clinicians. Setting the level at 34 μmol/L (2 mg/dL) includes the infants perceived as cholestatic by clinicians and harmonizes with the definition used by most researchers.

We argue that for HDFN-associated cholestasis, the criterion of a CB level exceeding 20% of the total bilirubin level is also appropriate, since the onset of cholestasis is early and coincides with high levels of unconjugated bilirubin. If this criterion is not applied, many infants not perceived as cholestatic by clinicians would be diagnosed.

Regardless of the definition, the incidence of cholestasis among infants with HDFN in our study is very high compared to the generally accepted figure of 1 out of 2500 in term infants [23]. The great majority of our study cases were detected very early, at a median age of 1.1 days, with just a few outliers. One patient first fulfilled the cholestasis criteria at 45 days of age without any cause other than HDFN being identified. For this infant, we hypothesize that the onset of cholestasis probably occurred earlier as the CB was not analyzed between 5 days of age, when it was 25 μmol/L, and 45 days when it fulfilled our criteria for cholestasis. In the absence of any consensus regarding the appropriate time window for attributing cholestasis to HDFN, we considered bilirubin tests within the first 90 days of life. This definition enabled us to be more inclusive in capturing cases and in detecting late effects on the liver from HDFN. Early diagnosis of cholestasis is important for correct management. If only total bilirubin is analyzed, conjugated hyperbilirubinemia may be missed and as a result, unnecessary phototherapy, directed at unconjugated hyperbilirubinemia, applied. Intensive phototherapy of a cholestatic newborn increases the risk of bronze baby syndrome [24]. In addition, appropriate cholestasis management, including prompt treatment with fat-soluble vitamins, may be delayed.

Prenatal treatment with IUTs and maternal alloimmunization with anti- D, -c or -K in combination with other red cell antibodies were independent risk factors for cholestasis in our cohort. In the study from the Netherlands, Smits-Wintjens et al. similarly found that D-immunization and IUT were independent risk factors for cholestasis [18]. However, the proportion of infants receiving IUTs was 66% in the Dutch study compared to 22% in our cohort, which might reflect more severe HDFN in the neonates admitted to the national Dutch referral center than in our population-based cohort. In our cohort, D-immunization alone was not an independent risk factor for cholestasis, but when we investigated the presence of other antibodies in combination with D-antibodies (or with c- or K-antibodies), we found highly significant associations with cholestasis. This is consistent with several studies that have reported that the presence of multiple antibodies, especially in combination with D-antibodies, may aggravate the severity of HDFN [4–7].

Postnatal exchange transfusions, immunoglobulin treatment and erythrocyte transfusions were more common in cholestatic than non-cholestatic infants in our cohort which also supports the hypothesis that a more severe HDFN is associated with cholestasis, as these treatments are more likely to be required in more severe cases of HDFN. This is also reflected in the difference in gestational age at birth and birth weight between cholestatic and non-cholestatic infants, since delivery is most often planned and induced earlier in high-risk pregnancies.

The early detection of cholestasis suggests that the hypothesis of inspissated bile syndrome alone cannot fully explain the pathogenesis of this condition, but may very well contribute postnatally. Regarding iron overload, too few non-cholestatic infants in our cohort had serum ferritin levels analyzed, and when available it was analyzed at different ages. However, we did note that ferritin levels were elevated in all infants with cholestasis where the test had been performed, and correlated strongly with the number of IUTs received prenatally, but also with the peak CB level. This is consistent with the hypothesis of other authors that iron overload may play a part in the pathogenesis [18]. However, elevated ferritin levels in neonates may also be secondary to liver injury [25]. The detection of cholestasis early in life suggests that the disease process in many cases may be initiated in utero. Although IUT is a strong independent risk factor for cholestasis, some infants develop cholestasis without receiving any IUT. Case-reports of cholestasis in non-immune mediated hemolytic conditions also raises the question of the hemolytic process itself and its effect on the fetal and neonatal liver [26–29].

The strength of this study is the population-based material, which provides an unbiased estimate of incidence of cholestasis. All pregnancies were managed in our tertiary center and there were no significant changes to the clinical management protocol during the study period. The study has several weaknesses. One limitation is the relatively small absolute number of infants who developed cholestasis, which limits the power for identification of independent risk factors. The available data on bilirubin levels was not collected as part of a study protocol, which may have led to overestimation of the age at onset of cholestasis in cases where CB levels were not monitored early or regularly enough. The latter could also have led to an underestimation of the true incidence of cholestasis. A future prospective multicenter study could overcome several of these issues. Since this study is retrospective, with relatively few cases and limited to one region in one country, generalization of the results should be made carefully. Nonetheless, the more severe cholestatic cases were most likely identified in our cohort, and can add to the clinical knowledge concerning this group of infants.

We propose that CB should be analyzed in infants with HDFN early after birth, along with total bilirubin, because of the high incidence of cholestasis in this group. Monitoring should be continued regularly to detect later onset and not terminated until normalization of CB levels is observed. Screening for cholestasis with CB within the first 2 days of life, and within the first week, would have discovered more than 60% and 80% respectively of the cases in our cohort.

We noted that cholestasis resolved in all cases. No infant had a diagnosis of chronic liver disease later in childhood. Thus HDFN-associated cholestasis, even though some cases may be very pronounced postnatally, seems to be a self-limiting condition in most cases. With regular follow-up and a clinical course that shows progress towards normalization of cholestasis in a child with HDFN, differential diagnostics with invasive, and possibly harmful, examinations could be reduced.

## Conclusions

We observed that cholestasis is common in infants with HDFN and present at birth or soon after in most cases. A more severe maternal alloimmunization with need for intrauterine transfusion is associated with increased risk for cholestasis. We propose that all neonates with HDFN should be routinely screened for conjugated hyperbilirubinemia to allow early detection of cholestasis and ensure correct treatment. Further research regarding cholestasis in infants with HDFN warrants a prospective and multicenter approach to gain enough scientific power for this rare condition.

## Supplementary information

Supplementary Figure 1

Supplementary Figure 2

Supplementary Table 1

## Data Availability

Data may be made available upon reasonable request to the corresponding author JT.
