# Supplementary figures and images for: Population-based incidence and risk factors for cholestasis in hemolytic disease of the fetus and newborn
Source: J Perinatol. 2022 Feb 22;42(6):702–7. doi: 10.1038/s41372-022-01345-1 (PMC9184269; doi:10.1038/s41372-022-01345-1)

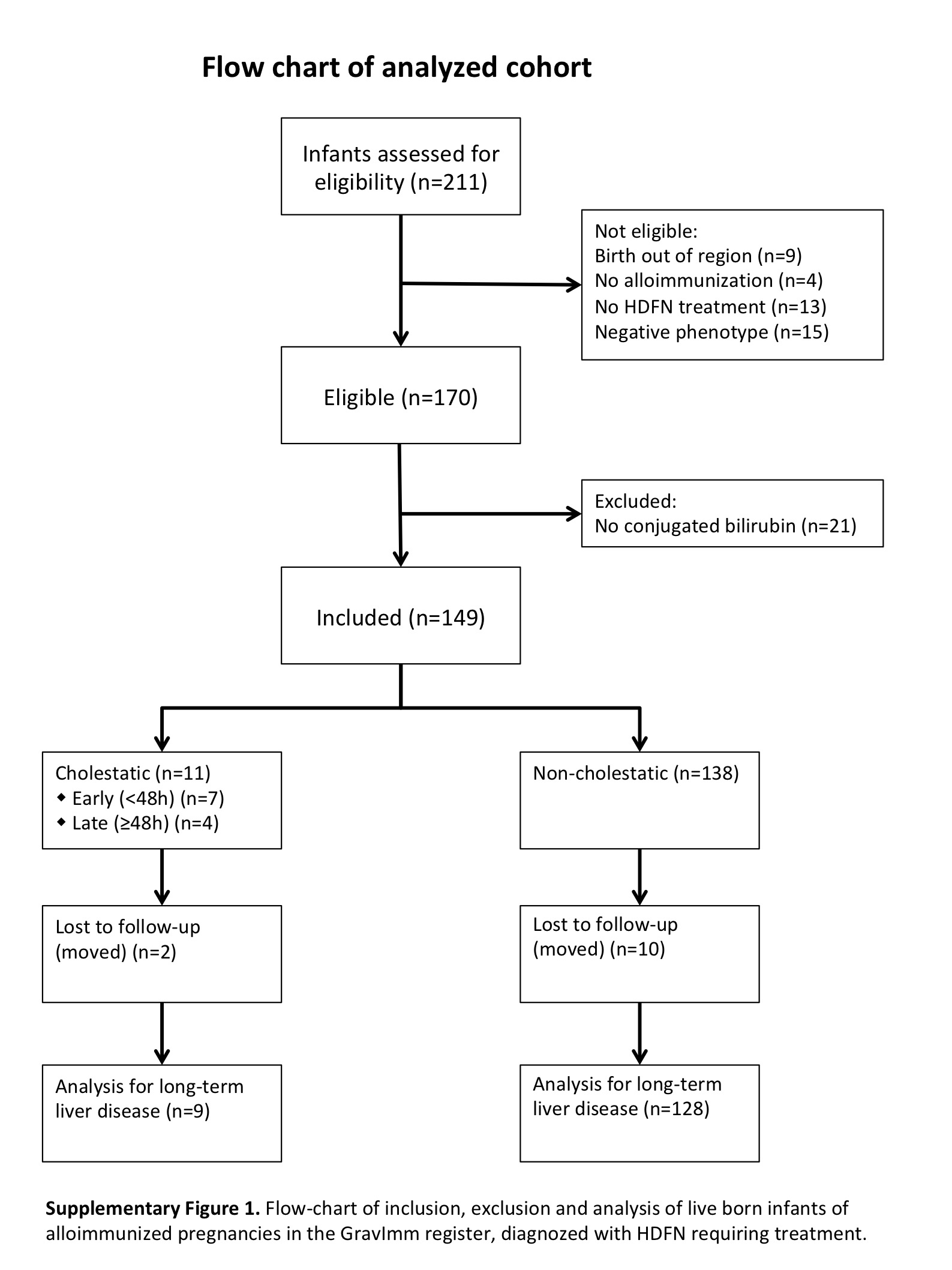

Supplement: Supplementary file 1 — Supplementary Figure 1 [file 41372_2022_1345_MOESM1_ESM.jpg]

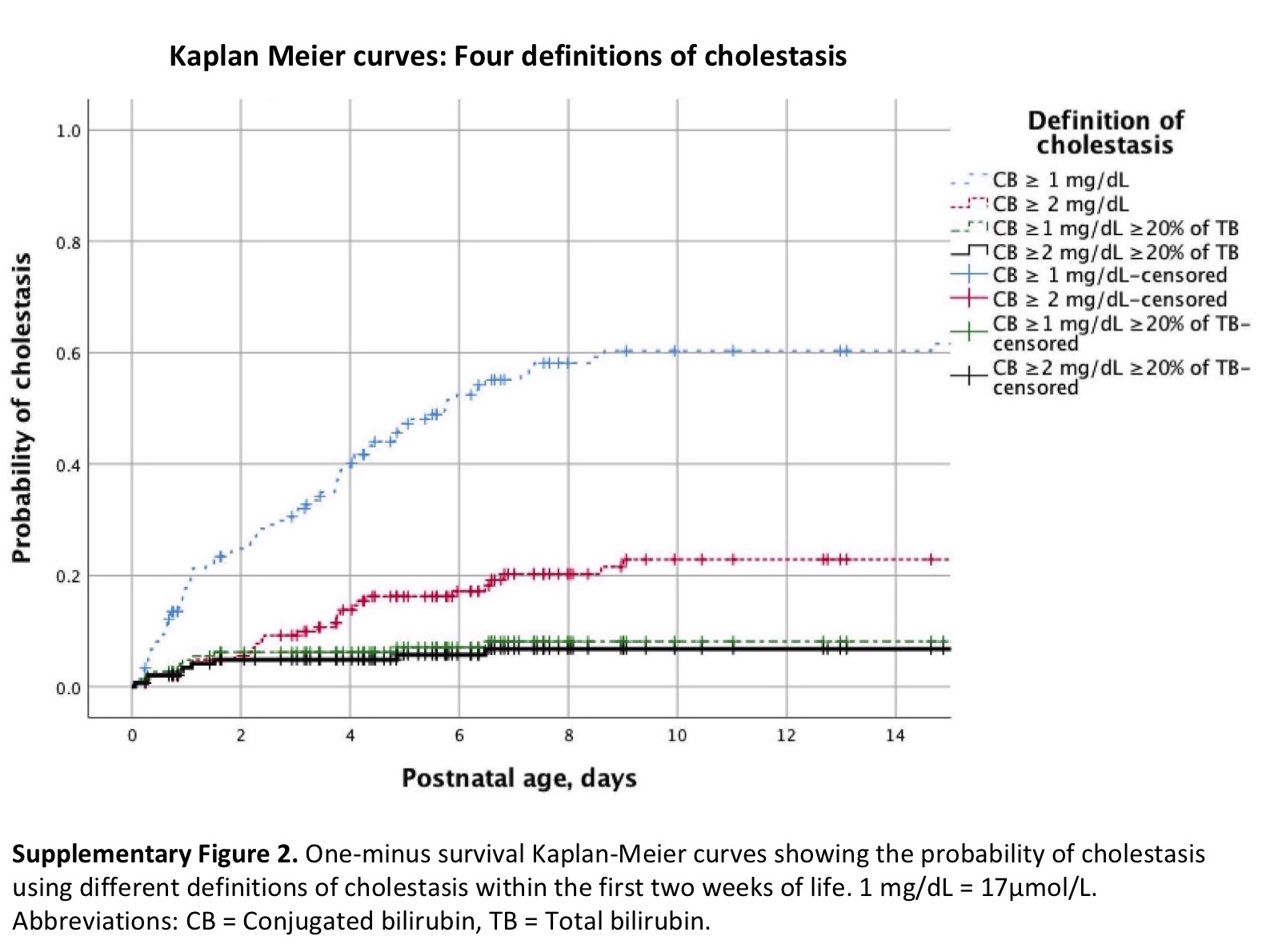

Supplement: Supplementary file 2 — Supplementary Figure 2 [file 41372_2022_1345_MOESM2_ESM.jpg]
