# Supplementary material for: Population-based incidence and risk factors for cholestasis in hemolytic disease of the fetus and newborn
Source: J Perinatol. 2022 Feb 22;42(6):702–7. doi: 10.1038/s41372-022-01345-1 (PMC9184269; doi:10.1038/s41372-022-01345-1)
Supplement: Supplementary file 3 — Supplementary Table 1 [file 41372_2022_1345_MOESM3_ESM.docx]

| **Supplementary Table 1.** Univariate Analysis of Association with Cholestasis | | |
| --- | --- | --- |
|  | **Crude OR (95% CI)** | **p** |
| Gestational age at birth (per additional week) | 0.75 (0.60-0.94) | 0.014* |
| IUT, at least one (ref no IUT) | 21.4 (4.3-105.3) | <0.001* |
| Birth weight, (per additional gram) | 0.999 (0.998-1.000) | 0.025* |
| Maternal age at birth, years | 0.94 (0.82-1.08) | 0.38 |
| Cesarean delivery (ref vaginal delivery) | 3.5 (0.98-12.79) | 0.055 |
| Female gender (ref male) | 1.1 (0.3-3.9) | 0.83 |
| D-immunization | 1.6 (0.4-6.4) | 0.50 |
| D-immunization, with multiple antibodies | 8.0 (2.2-28.9) | 0.002* |
| D, c or K immunization | 2.1 (0.3-17.2) | 0.49 |
| D, c or K immunization, with multiple antibodies | 12.7 (3.1-51.3) | <0.001* |
| Multiple antibodies of any types | 11.0 (2.7-44.1) | <0.001* |
| Any incompatibility in ABO-system | 0.68 (0.81-5.71) | 0.72 |
| Phototherapy, hours (per hour of treatment) | 0.998 (0.989-1.007) | 0.66 |
| Exchange transfusions (per transfusion) | 3.8 (1.8-7.8) | <0.001* |
| Exchange transfusion, any (ref no exchange transfusion) | 7.2 (2.0-26.4) | 0.003* |
| IVIG treatment, any (ref no IVIG treatment) | 4.4 (1.1-16.5) | 0.031* |
| Exchange transfusion AND/OR IVIG treatment, any (ref no treatment) | 7.0 (1.8-27.8) | 0.006* |
| Any parenteral nutrition (ref no parenteral nutrition) | 3.6 (0.7-19.6) | 0.14 |
| Abbreviations: OR, odds ratio; CI, confidence interval; IUT, intrauterine transfusion; IVIG, intravenous immunoglobulin. | | |
